# NON-FAMILIAL INTERGENERATIONAL NETWORK TIES: MOVING THE NEEDLE ON MEASUREMENT

**DOI:** 10.1093/geroni/igz038.2331

**Published:** 2019-11-08

**Authors:** Shannon E Jarrott

**Affiliations:** The Ohio State University, Columbus, Ohio, United States

## Abstract

GSA and other professional organizations recognize the threat that ageism poses to the country’s health and welfare, from its youngest to oldest citizens. Reframing Aging involves communication and outreach strategies to inform the conversation about aging and its implications. Non-familial intergenerational relationships can support the Reframing Aging initiative. By fostering positive, intentional, and mutually beneficial interactions, intergenerational exchange can achieve a variety of individual, relational, and community goals. Measures of their impact should reflect these goals; unfortunately, intergenerational network ties are typically represented by measures of young people’s attitudes towards older adults. Practitioners, researchers, policy makers, and funders need additional indicators to document the best practices and potential impact of non-familial intergenerational programs. Reflecting recent systematic surveys of measurements and 20 years of intergenerational research, the current paper addresses challenges of measuring non-familial intergenerational relationships and presents select measures appropriate for common network ties.

